# Using principal component analysis to explore consumers' perception toward quinoa health and nutritional claims in Gweru, Zimbabwe

**DOI:** 10.1002/fsn3.2071

**Published:** 2020-12-22

**Authors:** Tavagwisa Muziri, Paul Chaibva, Anyway Chofamba, Tendai Madanzi, Pepukai Mangeru, Nhamo Mudada, Shephard Manhokwe, Amiel Mugari, Diego Matsvange, Cyril Tapiwa Farai Murewi, Liboster Mwadzingeni, Raymond Mugandani

**Affiliations:** ^1^ Department of Agronomy Midlands State University Gweru Zimbabwe; ^2^ Department of Research and Specialist Services Plant Quarantine and Plant Protection Services Institute Mazowe Zimbabwe; ^3^ Department of Food Science and Nutrition Midlands State University Gweru Zimbabwe; ^4^ Sizimele – Action for Building Resilience in Zimbabwe Bulawayo Zimbabwe; ^5^ Department of Applied Mathematics and Statistics Midlands State University Gweru Zimbabwe; ^6^ School of Agricultural, Earth and Environmental Sciences University of KwaZulu Natal Pietermaritzburg South Africa; ^7^ Department of Land and Water Resources Midlands State University Gweru Zimbabwe

**Keywords:** health, nutrition, perception, principle component analysis, quinoa

## Abstract

Quinoa is one of the ancestral grains now considered as the “superfoods of the future” due to their characteristics as functional foods with great environmental adaptability. The Zimbabwe Resilience Building Fund has introduced quinoa in Zimbabwe to increase resilience of farming systems in light of environmental shocks. In order to improve adoption of the crop, it is important to understand the combined effects of socio‐demographic variables on consumer perceptions of the crop. However, there is limited literature on the combined effects of socio‐demographic variables on quinoa health and nutritional benefits in Zimbabwe. In this study, we used principle component analysis to identify consumers' perception about health and nutritional benefits of quinoa in Gweru Urban District, Gweru, Zimbabwe. A questionnaire was designed and applied to 200 participants. However, only 167 forms were valid for analysis. Age, education, and income level are main factors that determine consumer perceptions on health and nutritional claims. The results indicate that quinoa need to be promoted a as a crop that goes beyond food and income security by placing additional emphasis on health and nutritional aspects. This is very insightful in light of the need to improve the uptake of the crop by smallholder farmers in Zimbabwe. However, the use of self‐reporting survey method has received criticism for failing to get detailed information on perceptions of individuals. These findings could be handy to promote quinoa as a climate smart crop with additional health and nutritional benefits. This is one of the novel research studies exploring the motives of Zimbabwean consumers towards quinoa as a functional food crop. The work also contributes to knowledge on consumer food preferences.

## Graphical abstract

In this study, we analyzed the perceptions of consumers on quinoa health and nutritional claims in Zimbabwe using the prt6incipal component analysis. We observed that the participants' perceptions toward the health and nutritional claims of quinoa could be explained by a combination of various sociodemographic variables.

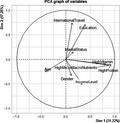

## 1 INTRODUCTION

Increasing agricultural productivity to realize food, health, and nutritional security is some of the major global challenges, particularly in developing countries. However, improving agricultural productivity in smallholder farmers of most African countries is hampered by unsustainable agricultural practices leading to a decline in soil organic matter, soil erosion, and soil nutrient mining. Increased rainfall variability in a changing climate aggravates these challenges. Consequently, a paradigm shift in cropping systems, supported by the United Nations agencies, is necessary to improve food, health, and nutritional security in these marginal environments in the face of a highly variable climate. The Food and Agricultural Organization (FAO) of the United Nations has identified quinoa (*Chenopodium quinoa)* as a “mother crop” with high nutritional value and enormous biodiversity (Bastidas et al., 2016) and capable of performing well in nutrient‐deprived soils and harsh climates. Therefore, it is not surprising that as a way of recognizing the good deeds of the Andeans in preserving this crop for food security, the United Nations General Assembly declared the year 2013 the “Quinoa International Year” (Bazile et al., 2015).

Quinoa offers a credible entry point to address the global food, health, and nutritional security concerns in the 21st century (Montemurro et al., 2019; Zikankuba & James, 2017). The crop is an exceptional “superfood of the future”, partly due to its functional characteristics (Singh et al., 2016). The functional foods are akin to traditional foods and provide certain health benefits in addition to offering basic nutritional services (Roberfroid, 2007).

Although quinoa has remained unpopular in most African countries, it has since been introduced in Ethiopia and Kenya in the 1990s, Malawi in 2010 (Gardner et al., 2019), and most recently (2017) in Zimbabwe by the Zimbabwe Resilience Building Fund (ZRBF). The ZRBF is supported by the Ministry of Lands, Agriculture, Water, Climate and Rural Resettlement (MLAWCRR), the European Union (EU), the Embassy of Sweden, the United Nations Development Programme (UNDP), and the UK Department for International Development (DFID). Its aim is to increase the capacity of at‐risk individuals, households, and communities to protect development gains and achieve improved well‐being in light of the harsh economic, environmental, and social shocks and stresses.

The production of quinoa in Zimbabwe is still at its infancy. However, research trials have been running at the Midlands State University (MSU) Farm since 2018 through the ZRBF. These trials' preliminary results indicate extremely promising yields in the range of 2–4.3 t/ha, depending on season and site conditions. These yields are comparable to the range of 1.585–2.097 t/ha obtained in Kenya using 24 varieties under irrigation (Murphy & Matanguihan, 2015). Two studies that evaluated the crop's yield potential under dryland conditions in Europe reported lower yields of 1.72 t/ha (Stikic et al., 2012) and 1.9 t/ha/ (Pulvento et al., 2010). Meanwhile, the preliminary results of ongoing trials at MSU show that the crop takes a maximum of 83 days after emergency to reach maturity. These promising results of quinoa productivity are very encouraging. Over 50 smallholder farmers from Matobo, one of the arid districts of Zimbabwe, have volunteered to pioneer the expanded production of quinoa under the ZRBF. Apart from trials at MSU, no other quinoa production activities noted in the country. Expanding these trials to multidistrict should guarantee more quinoa production data for the country. However, successfully upscaling and rolling out of the crop in smallholder farming systems of Zimbabwe depend on the acceptability of the crop by the general Zimbabwe populace. It is important to note that currently, quinoa's consumption in Zimbabwe is limited to the rich suburbs of Harare and Bulawayo cities, where processed quinoa grain was observed sparingly in selected upmarket supermarkets.

### 1.1 Health benefits of Quinoa

The crop has numerous health benefits with a unique lipid, fiber, micronutrient, and macronutrient profile. Its nutrient levels are often higher than those of cereal‐based products (Montemurro et al., 2019). It has a favorable omega‐6: omega‐3 ratio (Montemurro et al., 2019), which is perfect for decreasing the risk of cancer, cardiovascular, and inflammatory diseases (Mohyuddin et al., 2019; Vega‐Gálvez et al., 2010). It is also a good source of fiber, which is important for decreasing the risk of high blood pressure, diabetes, development of hemorrhoids, and weight control (Singh et al., 2016).

The crop is also rich in calcium, magnesium, potassium, phosphorus, sodium, iron, manganese, zinc, and copper (Filho et al., 2017). A good number of these nutrients are inadequate in resources poor households diets (Singh et al., 2016), especially in low‐ and medium‐income countries. The adequate supply of these minerals is particularly important in the prevention of hidden hunger in pregnant and lactating women, as well as children under five years and the elderly (Jacobsen et al., 2013; Shekhar, 2013). It could also offer a new perspective in Zimbabwe, where undernourishment, at 32.8% as of 2010–2012 (Murphy & Matanguihan, 2015), remains the top nutritional challenge (UNDP, 2017).

### 1.2 Nutritional benefits of Quinoa

Proteins are important in the human diet. Several publications have shown that quinoa has a relatively high protein content (; Mohyuddin et al., 2019; Srujana et al., 2019) of high biological value (Singh et al., 2016) (73%). Its protein content is comparable to that of beef (74%) but higher than all the cereals (Table 1) (Bastidas et al., 2016). Furthermore, quinoa has an unbelievable balance of all the essential amino acids (Jancurová et al., 2009; Satheesh & Fanta, 2018; Srujana et al., 2019; Zikankuba & James, 2017) necessary for human life (Filho et al., 2017), making it a complete protein source (Mohyuddin et al., 2019). According to the FAO and World Health Organization (WHO), quinoa satisfies the daily‐recommended intake of amino acid for adults. It might offer a cheaper source of nutrition, particularly for infants (Bastidas et al., 2016), who are normally weaned to starch porridges with poor nutritional composition. Meanwhile, the crop has higher levels of lipids and minerals compared to most cereals (Table 1). Thus, the crop, which provides affordable, high‐quality proteins (Bastidas et al., 2016; Filho et al., 2017), is beneficial to poor communities in developing countries (Zikankuba & James, 2017 **),** who normally consume cereal‐based products compared to animal and legume products.

**TABLE 1:** Composition of the quinoa grains in comparison with some cereals (g/100 g dry mass)

Component	Quinoa	Rice	Barley	Wheat	Corn	Rye	Sorghum
Lipids	7.9	3.2	1.3	2.8	5.3	1.8	3.6
Proteins	16.3	8.8	11.0	14.8	10.5	11.6	12.4
Ashes	2.7	1.7	1.2	1.8	1.3	1.8	1.7
Dietary fiber	7.0	3.5	15.6	10.7	7.3	15.1	6.3
Carbohydrates	74.0	86.3	86.5	80.6	82.9	84.3	82.3

Note

Source (Maradini‐Filho 2017).

Based on the aforementioned attributes, quinoa is a special candidate for improving food, health, and nutritional security in a changing climate. Its suitability for many diverse climates and soil conditions makes it an ideal crop for southern Africa and especially Zimbabwe considering the threats of climate change and decline in soil quality on crop yields (Thierfelder et al., 2015). However, as quinoa is a new crop, the perception of consumers toward the crop is important.

### 1.3 consumer perceptions

Consumers' perceptions of the health benefits of crops are important in understanding preferences (Meyerding et al., 2018) and evaluating the strength and weaknesses of the product in a market (Sabbe, 2009), particularly when opening up new product markets (Thome‐Ortiz et al., 2019).

An informed consumer aggregates knowledge about food from various available sources and compares it with the information on the product labels (Pinto et al., 2017). Therefore, the favorable perception of products accompanied by health claims depends on the relevance of the claim, product category, or ingredients, the production method involved to enrich the product (Stancu et al., 2017). Nonetheless, though nonsensory and sensory characteristics are important factor**s** in choosing foods (Pinto et al., 2017), the health and nutritional aspects are increasingly becoming important.

In general, sociodemographic variables, such as gender (Bower et al., 2003; Lyly et al., 2007; Sabbe, 2009); age (Bimbo et al., 2017; Thome‐Ortiz et al., 2019); marital status (Thome‐Ortiz et al., 2019); wealth status (Groeniger et al., 2017; Kaur & Singh, 2017); international travel (Sabbe, 2009; Verbeke & López, 2005); and education (Kaur & Singh, 2017), are important determinants of the preference for functional foods. Nevertheless, we know a lot about the importance of individual personal attributes, little is known about the combined effects of interrelated sociodemographic variables. For example, some studies have shown that a healthy dietary pattern is more common in married women of an elite social class, while younger and unmarried women of low socioeconomic class tend to exhibit dietary risk patterns (Ax et al., 2016; Bojorquez et al., 2015; Li et al., 2016).

Principal component analysis (PCA) is a multivariate approach that seeks to describe observations using intercorrelated variables as opposed to independent relationships, enabling the inference of confounding variables. PCA is relevant in such analysis since many of the confounding variables are difficult to identify and measure and at the same time extremely hard to control in many experiments. Traditional approaches in measurement of relationships where there are confounding factors such as multiple regression often results in questionable conclusions. The major aim of a PCA is to synthesize, reduce the size of data, and present it in new set of variables known as principal components (Abdi & Williams, 2010). PCA is extensively used in the derivation of dietary patterns and visualizing some hidden fundamental patterns in the observed data (Smith et al., 2013).

Therefore, the objective of this study was to investigate the hidden confounding sociodemographic variables related to the nutritional and health perception claims over quinoa in Gweru, Zimbabwe, using PCA.

## 2 MATERIALS AND METHODS

### 2.1 Study area

The survey was conducted in Gweru urban district, Midlands Province, Zimbabwe, between November 2019 and February 2020. Random sampling was employed in order to get participants for the study.

### 2.2 The questionnaire

Following a review of existing literature, appropriate items to measure the health and nutritional‐related consumption motives for quinoa were identified (Table 2). Specifically, the study adopted some of the nutritional and health statements from a previous study on consumer perception on amaranth by Rojas‐Rivas et al. (2019). The authors granted permission for the use of the survey tool. Our questionnaire was triangulated with focus group discussions and key informant interviews. However, the results of the focus group discussions and key informant interviews are not reported in the study.

**TABLE 2:** Quinoa health and nutrition claims

Topic	Motives for consumption
Promoting heath	Good to fight or prevent a disease
	Good for keeping me healthy
	Good for improving physical condition
	Good for weight control
Nutritional properties	Has a high protein content
	Has a high vitamins content
	It has a high content of micronutrients and macronutrients

Note

Statements adapted from Rojas‐Rivas et al. (2019) with minor adjustments.

1 “strongly disagree to” 5 “strongly agree.”

These, together with relevant documented sociodemographic factors: age, gender, education, marital, and wealth status (Rivaroli et al., 2020), and international travel experience, formed the study questionnaire. The gender of the respondents was measured using two categories, male and female. Age was measured using five categories, below 18; 18–25; 26–35; 36–45; and above 45. The marital status of the participants was measured using two categories, married and unmarried. Meanwhile, the level of education was measured using five categories, no formal education, primary level, secondary level, diploma, and degree or higher. We classified the respondents travel experience into two categories, no travel experience, and some travel experience.

### 2.3 Administering of Questionnaire and data recording

The questionnaire was pretested with 15 individuals before data collection for clarity and timing. Necessary adjustments were made where appropriate. Respondents provided informed consent prior to completing the questionnaire. No incentives or payments were made for participation. Before answering the questionnaire, the participants were asked to go through the health and functional mock package that contained a brief description and image of the crop, which accompanied the questionnaire. The participants were asked to respond to the health and nutritional statements on a five‐point Likert scale (strongly disagree to strongly agree). The pretested questionnaire was administered to a sample of 200 participants. However, during the recording and processing of data in SPSS, it was observed that only 167 responses were complete and suitable for in‐depth analysis. Data cleaning and coding were performed using SPSS version 18.

### 2.4 Data analysis

#### 2.4.1 Principal component analysis model for attitude, nutrition, and health variables

The patterns and influence of sociodemographic on nutritional or health variables were identified using PCA. The PCA is a statistical approach used to reduce large data sets into smaller data sets without losing much of the original sample data (Mwadzingeni et al., 2020; Vyas & Kumaranayake, 2006). Principal components (PC) differ in the number of variables with higher weighting, defining PC's characteristics. Meanwhile, variables with acceptable component loadings higher than ± 0.3 are regarded as highly correlated with a particular pattern (Mak et al., 2013). A screen plot is used to decide the variables to use in the final analysis. In this study, the data set comprising personal demographic, health, and nutrition data were analyzed in three stages. To achieve this, PCA was performed initially on the data set that excluded health variables to determine the factors responsible for nutrition. Similarly, nutrition factors were excluded from the dataset to determine factors affecting health. Lastly, all the variables were considered. The PCAs were performed using R software version 4.0.2.

#### 2.4.2 Principal component analysis model description

If an *i*‐dimensional variable with mean µ is defined according to Equation (1)

(1) $$X^{T}=\left[X_{1},\ldots ,X_{i}\right]$$

where *X* = factors, *i* = *i* ^th^ factor, and *X* ^T^ is the transpose of *X*.

To find a new set of variables, *Y*_1_,*Y*_2_,…,*Yp* (whose variance decreases from first to last, each *Y_i_* (principal components) is taken to be a linear combination of the *X_j_* (sociodemographic and either nutritional or health variables) as in Equation (2):

(2) $$Y_{j}=a_{1j}X_{1}+a_{2j}a_{2j}X_{2}+\ldots +a_{\mathrm{pj}}X_{p},$$

where $a_{j}^{T}=\left[a_{1j},\ldots ,a_{\mathrm{pj}}\right]$.

## 3 RESULTS

### 3.1 Characteristics of participants

Table 3 shows the results of the sociodemographic profile of the respondents. Although not necessarily in the majority, a greater proportion of the participants were in the 36‐ to 45‐year bracket. In terms of gender, a majority of the participants were female, while the remainder were male. The results also reveal that a majority of those who completed the survey was married while the remainder was unmarried. A majority of the respondents classified themselves as being moderately poor. Meanwhile, a majority of the respondents had traveled beyond the Zimbabwean border. With reference to schooling, close to 1% of the participants had no formal education. In comparison, slightly less than 50% had continued schooling beyond secondary/advanced level education, of which about one‐fifth had attained at least a first degree.

**TABLE 3:** Sociodemographic variables of the study participants

Variable	Description	Frequency	% share of sample
Gender	Female	87	52.1
	Male	80	47.9
Age	<18	2	6
	18–25	28	16.8
	26–35	69	41.3
	36–45	39	23.4
	>45	30	18
Marital status	Unmarried	66	39.5
	Married	101	60.5
Education	No formal schooling	24	14.4
	Primary	12	7.2
	Secondary	42	25.1
	Diploma	35	21.0
	Degree or higher	54	23.2
External travel	No experience	70	41.9
	Experience	97	58.1

### 3.2 Interpretation of PCs concerning sociodemographic, variables nutrition, and health factors

**FIGURE 1:**
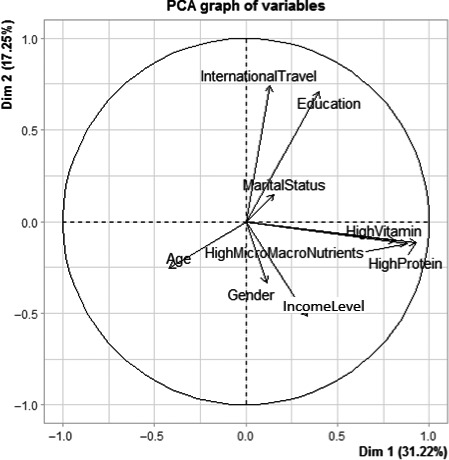
Biplot of the variables excluding global health

**FIGURE 2:**
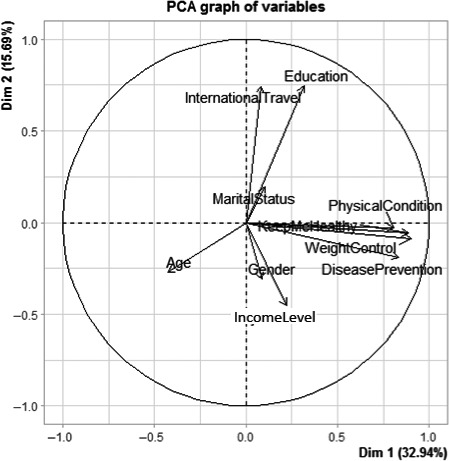
Biplot of the variables excluding global nutrition

**FIGURE 3:**
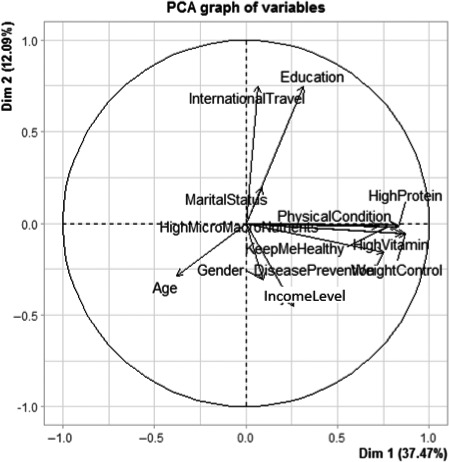
Biplot of all the variables

Pattern analysis of the variables used in the questionnaire was performed for the nutritional and healthy aspects and all variables. Table 4 shows the eigenvalue, the proportion of the retained principal components, and loadings patterns corresponding to each of the three cases considered in the principal component analysis. For each of the nutrition and health claims and all variables obtained from the questionnaire, retained components (eigenvalue > 1, criterion) explained 61.49%, 60.80%, and 68.01%, respectively. Proxy biplots for these are shown in Figures 1, 2, 3, respectively.

**TABLE 4:** Eigenvalues, percentage of variance, and the component loadings for the patterns retained for each of nutrition measures, health measures, and all variables

	Nutrition			Health			All variables			
	PC1	PC2	PC3	PC1	PC2	PC3	PC1	PC2	PC3	PC4
Eigenvalue	1.6762	1.2461	1.0823	1.8151	1.2524	1.1032	2.2069	1.2537	1.1165	1.0736
% of variance	0.3122	0.1725	0.1302	0.3294	0.1568	0.1217	0.3747	0.1209	0.0959	0.0887
Component loading for each pattern										
Age	0.2747	0.0747	0.2075	**0.3258**	0.0017	0.1053	0.2084	0.1369	0.1669	**−0.5380**
Gender	−0.0107	0.0791	−0.0153	−0.0006	0.0784	−0.0230	−0.0169	0.0731	−0.0271	0.0006
Marital status	−0.0437	−0.0106	−0.1037	−0.0444	−0.0084	−0.0944	−0.0253	−0.0278	−0.0957	0.0418
Education	**−0.5193**	**−0.7053**	**−0.3434**	**−0.5907**	**−0.6538**	**−0.3468**	**−0.3059**	**−0.8222**	**−0.3433**	−0.0411
Major income	−0.2184	**0.5894**	**−0.7432**	−0.1432	**0.5833**	**−0.7715**	−0.1796	**0.4642**	**−0.8233**	0.0869
International travel	−0.0588	−0.1550	0.0011	−0.0717	−0.1489	−0.0016	−0.0263	−0.1628	0.0022	−0.0207
High protein	**−0.5012**	0.2300	**0.3615**				**−0.4251**	0.1025	0.0504	**−0.4433**
High micro macro nutrients	**−0.4041**	0.1940	**0.3097**				**−0.3243**	0.0796	0.0044	**−0.4679**
High vitamin	**−0.4327**	0.1690	0.2215				**−0.4053**	0.0855	0.0999	−0.0906
Disease prevention				**−0.3725**	**0.3163**	0.2757	**−0.3142**	0.1542	0.1940	**0.3689**
Keep me healthy				**−0.3437**	0.2010	0.2903	−0.2717	0.0581	0.2206	**0.3424**
Weight control				**−0.3343**	0.1891	0.2885	−0.2997	0.0690	0.2397	0.1469
Physical condition				**−0.3855**	0.1663	0.1429	**−0.3415**	0.0334	0.1009	0.0242

Bold values indicate components that are highly corelated with a particular pattern.

#### 3.2.1 Factors affecting respondents' quinoa uptake toward nutritional claim

The first principal component revealed higher negative correlations with all three nutrition measures: high protein, high vitamin, and mineral content (both micro‐ and macronutrients). Education also contributes negatively to this first pattern. The second component depicts a strong positive relationship with the level of income and education. Level of income, education, high protein, high mineral (both micro and macronutrients) characterize the third pattern.

The three nutrition measures are approximately related, as indicated in the biplot (Figure 1) between the first two principal components. These variables are closely packed together, and their arrows have similar lengths. International travel, education, and level of income seem to also contribute to the first two components. However, international travel is at an angle of almost 90 degrees to the nutritional proxy variables.

#### 3.2.2 Factors affecting respondents' quinoa uptake toward health claims

Table 4 shows the results of the PCA for sociodemographic variables affecting a positive attitude toward health claims. Pattern 1 describes the negative correlation of disease prevention, keeping health, weight control, physical condition, and education. However, age is positively related to the first pattern. Principal components 2 and 3 are mainly correlated with education and level of income.

The proxy biplot (Figure 2) between the first two principal components reveals that all the four health measures tend to be closely related to each other (all clustered together and having similar arrow lengths). International travel, education, and level of income seem to contribute to the first two components. Again, international travel makes an angle approximately make 90 degrees to the health proxy variables.

#### 3.2.3 Factors affecting respondents' quinoa uptake toward both health, nutrition, and all variables

Table 4 shows the relatedness of all the variables toward quinoa uptake in Gweru. Pattern 1 describes a group of individuals who consider all the three nutrition measures (high vitamin, high protein, and high mineral), disease prevention, physical condition, and education. Level of income is dominant in patterns 2 and 3. Meanwhile, pattern 4 strikes a balance between nutrition (high protein and high minerals) and health (disease prevention and keeping healthy) measures.

The proxy biplot (Figure 3) between the first two principal components reveals that both nutrition and health measures are approximately related as reflected by the clustered arrows' almost equal lengths. The variation of these variables is also closely similar, as shown by the length of the arrows. Figure 3 also reveals that international travel and education seem to be closely related as they are close to each other and have similar arrow lengths. The same is observed in both Figures 1 and 2. However, in all the boxplots, the international travel vector seems to make a right with both the nutrition and health claims variables. This indicates that they are no correlation between one's experience in international travel and the claims proxies.

## 4 DISCUSSION

### 4.1 Description of study participants

This study was the first to use a survey tool to decipher consumers' perceptions on quinoa nutritional and health claims in Zimbabwe. The results revealed that a greater proportion of the participants were between the age of 36–45 years (Table 3). According to the recent (2012) census in Zimbabwe, a greater proportion of the population is between 36 and 45 years old (ZIMSTAT, 2012). Further to this, a greater proportion (52.1%) of the respondents was female (Table 3). These results are consistent with findings by ZIMSTAT (2012). In terms of marital status, the results show that the majority of the participants were married, which is also consistent with findings by ZIMSTAT (2012). Slightly below two‐thirds of the participants were moderately wealthy, while a majority had traveled beyond the Zimbabwean border.

### 4.2 Relationship between sociodemographic factors

#### 4.2.1 Relationship between sociodemographic factors and nutrition

The first component explains the nutrition factors (high protein, high minerals, and high vitamins). Attitudes of these urban‐based individuals are approximately highly dependent on the nutritional aspects. Education also played a role in this pattern. According to a study done in Nigeria, married and educated people are generally stable (Ojukwu et al., 2016). However, their poor financial status distorts their stability (Barbarin & Khomo, 1997). The level of income is key to access to better health services. The level of income influenced both patterns 2 and 3.

#### 4.2.2 Relationship between sociodemographic factors and health

Health benefits also seem to contribute highly to pattern 1. The urban population is sensitive to health provisions. Age and education also played a part in health issues. The second and third components were composed mostly of personal sociodemographic factors. Educated, married people were likely to travel to pursue greener pastures and so seek foods with health benefits. Moreover, education contributes to improved functional health literacy (Baker et al., 2000).

#### 4.2.3 Relationship between sociodemographic factors and all variables

The major determinants are based on nutrition and health factors, as revealed in component one. Both the second and third patterns explained personal sociodemographic factors. Respondents who are highly educated and at the same time earning high income have a higher nutritional literacy.

### 4.3 Strengths of the study

A strength of the current study stems from the use of previously established statements and scales with minor adjustments where possible in the survey, thereby enhancing the strength of the results. For example, the nutritional and health statements were from Thome‐Ortiz et al. (2019).

### 4.4 Limitations of the study

The limitation of the current study is the use of the self‐reporting survey method particularly where consumers were asked to look at the nutritional and health claims before completing the questionnaire. However, shoppers hardly use labels when shopping (Leathwood et al., 2007). Meanwhile, the sample size is limited to affirm that the study is representative of the study population. The study was undertaken in an urban environment, where there is high exposure to health, nutrition, and dietary promotional campaigns and easy access to information via different multimedia systems. It becomes notoriously difficult to generalize these findings to the whole country, in light of the fact that 67% of the total population in the country resides in rural areas. Future research could explore the possibility of employing word associations in studying consumer motives and reasons for eating certain food items in addition to the use of a more representative sample. Future studies in Zimbabwe need to investigate consumers' perception toward quinoa anthropometric, biochemical, physical, nonsensory, and sensorial attributes.

## 5 CONCLUSION AND RECOMMENDATIONS

Quinoa is a unique crop that can address such multiwhammy problems of soil fertility decline, lack of access to agricultural inputs, poverty, food insecurity, vulnerability to climate change, poor nutrition in infants and the elderly, obesity, cancer, and cardiovascular diseases. The promotion of quinoa as a crop that goes beyond the focus on food and income security by placing additional emphasis on health and nutritional aspects may improve its uptake by smallholder farmers in Zimbabwe and other African countries. The study concluded that the perception toward health and nutritional claims could be predicted since most of the factors/determinants are health‐related. The respondents from an urban setting make their choices basically relying on their improved functional health literacy. However, the other personal demographic parameters, especially education and level of income, play a secondary role. Nutritional claim for Quinoa relates to the educational level and income level of respondents in Gweru urban. The health claim of Quinoa relates to the age, education, and income level of respondents.

In contrast, both health and nutritional claim for Quinoa relates to the age and education of respondents. Based on these results, we concluded that there is huge potential for “Andeanizing the diet” among various consumers in Gweru. This is the inclusion of quinoa into the diets of individuals. The current study opens an avenue for further research on the need to make use of simpler statements on food packages to cater for the average consumer.

## CONFLICT OF INTEREST

The authors declare that they do not have any conflict of interest.

## ETHICAL APPROVAL

“This study does not involve any human or animal testing” and was approved by the Midlands State University Research Board”.

## INFORMED CONSENT

Written informed consent was obtained from all study participants.

## Data Availability

“The data that support the findings of this study area available from the corresponding author upon reasonable request”.
